# Dental Luting Cements: An Updated Comprehensive Review

**DOI:** 10.3390/molecules28041619

**Published:** 2023-02-08

**Authors:** Artak Heboyan, Anna Vardanyan, Mohmed Isaqali Karobari, Anand Marya, Tatevik Avagyan, Hamid Tebyaniyan, Mohammed Mustafa, Dinesh Rokaya, Anna Avetisyan

**Affiliations:** 1 Department of Prosthodontics, Faculty of Stomatology, Yerevan State Medical University after Mkhitar Heratsi, Str. Koryun 2, Yerevan 0025, Armenia; 2 Conservative Dentistry Unit, School of Dental Sciences, Health Campus, Universiti Sains Malaysia, Kubang Kerian, Kota Bharu 16150, Kelantan, Malaysia; 3 Department of Conservative Dentistry & Endodontics, Saveetha Dental College & Hospitals, Saveetha Institute of Medical and Technical Sciences University, Chennai 600077, Tamil Nadu, India; 4 Department of Orthodontics, Faculty of Dentistry, University of Puthisastra, Phnom Penh 12211, Cambodia; 5 Center for Transdisciplinary Research, Saveetha Dental College, Saveetha Institute of Medical and Technical Science, Saveetha University, Chennai 600077, Tamil Nadu, India; 6 Department of Histology, Yerevan State Medical University after Mkhitar Heratsi, Str. Koryun 2, Yerevan 0025, Armenia; 7 Department of Science and Research, Islimic Azade University, Tehran 1477893855, Iran; 8 Department of Conservative Dental Sciences, College of Dentistry, Prince Sattam Bin Abdulaziz University, Al-Kharj 11942, Saudi Arabia; 9 Department of Clinical Dentistry, Walailak University International College of Dentistry, Walailak University, Bangkok 10400, Thailand; 10 Department of Therapeutic Stomatology, Faculty of Stomatology, Yerevan State Medical University after Mkhitar Heratsi, Str. Koryun 2, Yerevan 0025, Armenia

**Keywords:** dental material, dental cements, luting agents, glass-ionomer cement, resin cement

## Abstract

The cementation of indirect restoration is one of the most important steps in prosthetic and restorative dentistry. Cementation aims to bond the prosthetic restoration to the prepared enamel or enamel and dentine. Successful cementation protocols prevent biofilm formation at the margin between tooth and restoration and minimize mechanical and biological complications. With the advancements in dental cements, they have been modified to be versatile in terms of handling, curing, and bond strengths. This review presents updates on dental cements, focusing on the composition, properties, advantages, limitations, and indications of the various cements available. Currently, dental restorations are made from various biomaterials, and depending on each clinical case, an appropriate luting material will be selected. There is no luting material that can be universally used. Therefore, it is important to distinguish the physical, mechanical, and biological properties of luting materials in order to identify the best options for each case. Nowadays, the most commonly used dental cements are glass-ionomer and resin cement. The type, shade, thickness of resin cement and the shade of the ceramic, all together, have a tangible influence on the final restoration color. Surface treatments of the restoration increase the microtensile bond strength. Hence, the proper surface treatment protocol of both the substrate and restoration surfaces is needed before cementation. Additionally, the manufacturer’s instructions for the thin cement-layer thickness are important for the long-term success of the restoration.

## 1. Introduction

Dental luting cements can be classified according to their chemical composition and application [1]. Regardless of the selected material, they should present consistency and film thickness compatible with cementation [2,3,4]. Dental cement can be oil-, water-, or resin-based [1,5].

Currently, there are numerous provisional and long-term cements available, and they differ from each other in their chemical composition, properties, and clinical applications. Provisional cements are usually oil-based or oil-free [1,5]. Previously, most of them contained eugenol, while nowadays they are mainly produced without it. These cements have weaker physical properties and greater film thickness than water- and polymer-based cement. Residual provisional cements should be thoroughly removed from the tooth before the application of final cements [6,7]. The presence of an oil component is being reduced since it can affect the curing process of long-term cementation, reducing the bond strength and justifying the use of eugenol-free cement [8,9,10,11].

Long-term cements present water-based or resin-based compositions [12]. Each of them has its own indication. Water-containing luting cements usually undergo an acidic reaction of solidification and become acidic when handling [13]. These cements are non-adhesive or may have low bonding strength to the tooth’s hard tissues.

The proper choice of cement and its precise application in clinical practice requires an awareness of the material composition, bonding mechanisms, and interactions with other restorative materials [14,15]. Therefore, the purpose of this review is to present an overview of dental luting cements’ properties, advantages, disadvantages, and indications, as well as to give recommendations on daily practice.

## 2. Selection of Luting Agents

**Figure 1:**
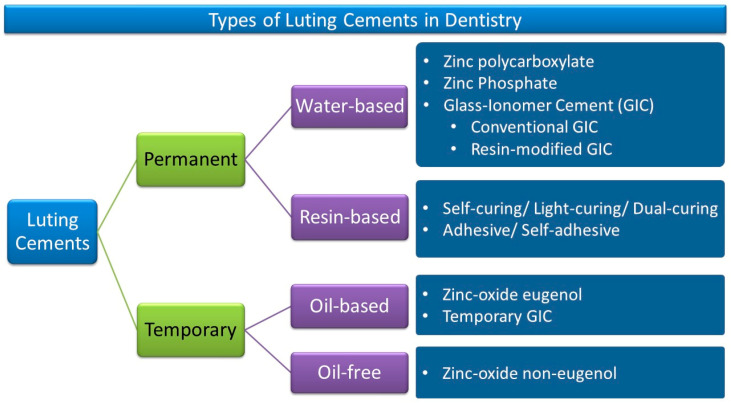
Various luting cements in dentistry with common examples.

The various types of luting agents with common examples are shown in Figure 1 [1,5,16,17,18]. Water-based luting cements release fluoride, such as glass-ionomer, resin-modified glass-ionomer, and zinc phosphate and zinc polycarboxylate cements [17], while resin cements are chemically similar to composite resins, which provide maximum strength to the tooth and indirect restoration when bonded with dental adhesives [19,20]. In addition, the surface etching of the restoration can provide micromechanical retention with resinous cement. Despite the physical properties of these cements, they are usually more sensitive to the cementation process [5,21]. Namely, metal copings, frameworks, or partial restorations are usually fixed with water-based cements, while composite cements are indicated when it is necessary to provide stronger adhesive bonding between the dental structure and restorative material [22].

There are different performances between cements and even between different manufacturers that claim to produce the same product. Therefore, before applying the luting cements, it is extremely important to follow the manufacturer’s instructions for use [16], as well as to perform all the suitable surface treatments on the restoration and substrate. Cements can also be divided into two groups: adhesive and non-adhesive (Table 1). Non-adhesive cements provide mechanical retention and are commonly based on water and reactive filler, while adhesive cements form an adhesive bonding with both tooth hard tissues and restoration, they consist of anhydrous-silanized non-reactive fillers [7,21].

The ideal dental cements maintain and protect the tooth’s hard structures, are highly resistant to tensile and compression stresses, fatigue resistant, and mechanically stable, present low shrinkage and strong bond strength to the tooth tissues and dental biomaterials, and prevent the development of caries in the adhesive interface. The basic properties of various dental luting agents are shown in Table 2. Ideally, the dental cements should be biocompatible, possess antimicrobial activity, provide marginal sealing, create a minimum film thickness, be easy to apply, be less soluble, present translucency and radiopacity, and have optimal working and curing time. In addition, they must have high fracture strength, optimal wettability (small wetting angle), and sufficient viscosity for complete spreading, as well as be esthetic when used in combination with a restorative material. Moreover, removing the excess material should be easy [5,16,22]. 

The cementation steps are as remarkable as other aspects of restorative dentistry since the incorrect choice of cement can result in impaired marginal integrity, esthetic issues, and malocclusion [23]; moreover, any wrongly performed step could compromise the final bond strength. The cement selection depends on the preparation type and restorative material that will be cemented [24]. Conventional cements were commonly used for metal alloys and fixed partial dentures. Zinc phosphate luting cements have been applied for many years. However, in more invasive preparations or for patients with a history of pulp hypersensitivity, a more biocompatible cement, e.g., polycarboxylate, should be used [16,25]. Some restorations require adhesive systems that are characterized by more complex applications [23,24]. In general, the stability and longevity of luting cements are not always predictable, as cement dissolution can lead to marginal caries. These problems are less pronounced with the use of resin-based and glass-ionomer cements due to their more predictable adhesive bonding and strength [23,26]. Furthermore, resin cements are especially preferable when the tooth is prepared following the principles of minimally invasive dentistry and all preparation margins are accessible [16]. The crowns cemented with zinc phosphate, polycarboxylate, and glass-ionomer cements can be cautiously removed in case of necessity, reducing the risk of damaging the prepared tooth. However, in order to remove a restoration adhesively cemented, it must be sectioned.

**Table 2:** Basic properties of various dental luting agents [16,22,27].

Luting Agent	Film Thickness (I *: Low)	Working/ Setting Time (min) (I *: Long/ Short)	Compressive/ Tensile Strength (MPa) (I *: High/High)	Elastic Modules (GPa) (I *: Dentine = 13.7; Enamel = 84–130)	Pulp Irritation (I *: Low)	Solubility (I *: Minimal)	Microleakage (I *: Very Low)	Color Stability and Aesthetics (I *: High)
Zinc phosphate	≤25	1.5–5/5–14	48–133/0.65–4.5	19.8	Moderate	High	High	Low
Zinc polycarboxylate	<25	1.75–2.5/5–9	57–99/1.4–6.3	16.1–19.5	Low	High	High to very high	Low
Conventional GIC ^F^	<25	2.3–5/6–9	93–226/2.36–5.3	11.2	Moderate	Low	Low	Low
RMGIC ^F^	>25	2–4/2–4	85–126/2.53–24	6.8	Moderate to high	Very low	Very low	Moderate
Resin cement	>25	1–5/1–7	52–224/5.07–41	11.8–16.5	Moderate to high	Very low	Very low	High

I *—ideal; ^F^—fluoride release, GIC—glass-ionomer cement, and RMGIC—resin-modified glass-ionomer cement.

## 3. Long-Term Luting Cements

**Figure 2:**
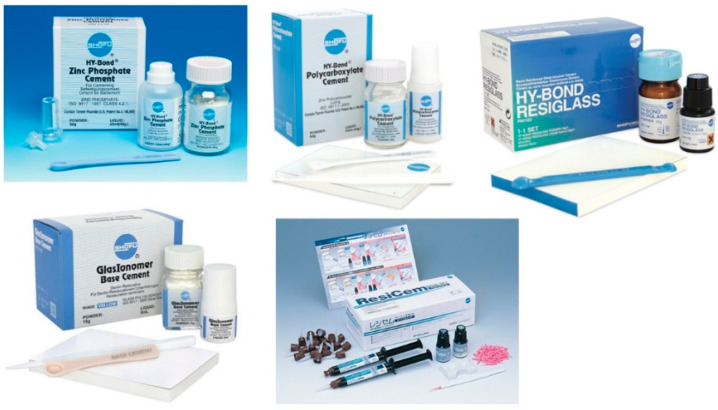
Long-term term cements (zinc phosphate cement, zinc polycarboxylate cement, glass-ionomer cement, resin-modified glass-ionomer cements, and resin cement) from Shofu company.

Long-term term luting cements include zinc phosphate cement, zinc polycarboxylate cement, glass-ionomer cement (GIC), resin-modified glass-ionomer cements (RMGICs), and resin cement (Figure 2). Table 3 shows the indications, retention, advantages, and precautions of various dental luting agents [22,27].

### 3.1. Zinc Phosphate Cement

The use of zinc phosphate cements began in 1878. It was the “gold standard” for fixing indirect restorations for many years, and it is still used for the same purpose. It has high compressive strength and reasonable working time. In addition, many years of clinical success allowed zinc phosphate cements to be considered with clinically acceptable effects when the material is properly handled. Zinc phosphate cements are generally used for the cementation of full metallic and metal–ceramic crowns and partial FDP, MCC with porcelain margins, and slip-cast alumina crowns. In addition, applying varnishes on prepared teeth reduces pulp sensibility, but at the same time, it reduces mechanical retention [16]. This luting thickness is approx. 25–40 microns, ensuring proper cement-layer thickness that facilitates cement flowability and provides some thermal insulation when metal crowns are used [28,29]. Moreover, zinc phosphate cements provide great mechanical retention [30,31,32].

Disadvantages of this water-based material are high solubility in oral fluid, low viscosity, low tensile strength, lack of an anti-cariogenic effect, and potential for hypersensitivity due to initially low pH [31,33]. The acidic pH (equal to 2) at the time of cementation can cause inflammation of the pulp tissue; while after fully hardening, the cement pH is 4.5–5.0. However, this information is not a consensus in the literature [31,34]. 

### 3.2. Zinc Polycarboxylate Cement

The era of dental adhesives began in the late 1960s, and the advent of polycarboxylate cements followed this development. These luting cements have higher tensile strength than zinc phosphate cements, but the compressive strength after 24 h is lower (55–85 MPa). Zinc polycarboxylate is generally used for the cementation of full metallic and metal–ceramic crowns and partial FDP, MCC with porcelain margins, casting on the patient with a history of post-treatment sensitivity, slip-cast alumina crowns, and cast post and core (metal), and aesthetic postcore and core (fiber and ceramic).

One of the advantages of these luting cements is the relative biocompatibility due to the large size of the polyacrylic acid molecules that cannot penetrate the dentine tubules. In addition, zinc polycarboxylate luting cements have a specific chemical adhesion to the tooth because they create chelating bonds with calcium. Therefore, these cements can be bonded to enamel and dentin (Table 2). However, due to its high viscosity, this material is difficult to handle [35,36,37].

Despite the advantages, longevity can be a problem for restorations cemented with polycarboxylate cements [38]. When the powder-to-liquid ratio suggested by the manufacturer is followed, zinc polycarboxylate cement can be extremally viscous. However, these cements have different rheological qualities (the flowability of materials), which leads to liquefaction when high forces are applied. This means that, despite their external viscosity, they can form a very thin layer. If the dentist unreasonably modifies the powder-to-liquid ratio, the solubility of the luting agent can increase by three times, which is a frequent cause of clinical failures [39,40]. 

The working time (2.5 min) is considered shorter than zinc phosphate (5 min), which can be troublesome in cementing multiple restorations. The residual amount is also more difficult to remove compared to zinc phosphate. Therefore, the excess should be removed before the resin phase or after curing. During the intermediate phase, the elastic curing phase, if the excess cement is removed, a considerable amount might displace from the restoration margin, causing a margin defect [41]. In addition, these cements have relatively high solubility. According to in vitro data, zinc polycarboxylate luting cement provides less retention than zinc phosphate cements. For this reason, the choice of this cement should be limited to preparations that have proper retention and stability [18,42,43,44]. 

### 3.3. Glass-Ionomer Cement

Glass-ionomer cements have been widely used as a restorative material since the 1970s, then gradually, they started to be used as a luting agent as well. This dental material has good compressive strength and tensile strength (Table 2). It has a low thermal expansion coefficient, and its adhesion to tooth tissues is comparable to polycarboxylate cements [45]. GICs are also generally used for the cementation of full metallic and metal–ceramic crowns and partial FDP, MCC with porcelain margins, slip-cast alumina crowns, metal posts, inlays, implant-supported crowns and bridges, and aesthetic postcore and core (fiber and ceramic). The existing variety of adhesive materials might be confusing for a dentist, and the choice of optimal cement is sometimes rather difficult. However, adhesive materials and methods have been significantly improved by optimizing restoration processing with minimal preparation [46,47,48].

These cements are available as a powder–liquid that should be mixed before their use in the oral cavity. The powder is mostly composed of calcium fluoroaluminosilicate glass (10–16% by mass is fluorine), while the liquid part is usually an aqueous solution of polyacrylic acid copolymers with itaconic or tartaric acid, as well as malic acid [16]. However, some manufacturers add polyacrylic acid and copolymer in the powder, and the liquid is composed of water or tartaric acid solution [45,49].

GICs are less soluble than zinc phosphate cements and release fluoride ions, which penetrate into tooth tissues, contributing to the remineralization of tooth tissues with an anti-caries effect [50,51]. After curing, GICs also exhibit bacteriostatic properties [16,50]. The retention of GIC was 65% higher than those of zinc phosphate cements [16]. It has good working properties and differs from zinc phosphate cements in its pronounced semi-opacity, which is good when it is used to restore the ceramic labial margin [46,52,53,54].

Despite the reported promising properties, GICs still have some drawbacks. They have low pH (of about 3,5), which can be associated with some discomfort due to hypersensitivity after bonding. Postoperative hypersensitivity was reported as a side effect of dentin dehydration or bacterial contamination rather than a cement-induced response. According to the literature, pulpal hypersensitivity is not associated with zinc phosphate and GIC when the manufacturer’s instructions are strictly followed [46,47,48].

In general, GICs are superior to zinc phosphate and zinc polycarboxylate cements in terms of mechanical properties, but their strength is reduced by early exposure to moisture because water changes the mechanical properties of GIC. It washes away cement-forming cations, and absorption occurs, resulting in erosion. GICs are also subject to significant erosion during the initial setting period. On the other hand, over-drying induces shrinkage that leads to the formation of cracks and hypersensitivity. For this reason, the marginal area of the restoration should be protected from exposure to liquids using varnish or petroleum-jelly-based products during the early period of the setting.

### 3.4. Hybrid Ionomer Cements or Resin-Modified Glass-Ionomer Cements

Glass-ionomer cements can be divided into two types: conventional and resin-modified glass-ionomer cements (RMGICs). Both types have similar mechanisms of adhesion, adhering to the tooth surface after forming ionic bonds due to the chelating between the carboxyl groups of the cements and the calcium and phosphorus of the dentin and enamel apatite. However, the bond strength of RMGICs to dentine is higher due to their composite part, while the bioactive effect is lower [55,56,57]. RMGICs were introduced in the early 1990s, and they have optimal physicomechanical properties, creating a strong bond with enamel and dentin [58,59,60,61]. RMGICs are indicated to retain total crowns and bridges, metal–ceramic crowns and bridges, zirconia frameworks and restorations, metal posts, metal inlays, orthodontic appliances, and aesthetic postcore and core (fiber and ceramic).

RMGICs have uses that are similar to GIC. However, they are characterized by high fracture resistance and greater wear resistance compared to conventional GIC. During this material’s development, the strength and insolubility of polymers have been combined with the fluoride-releasing and adhesive properties of GIC. These materials differ from resin cements because they are water-based and partially polymerized by a reaction between polyacrylic polymer and calcium fluoroaluminosilicate glass particles [55,56,57].

These materials are less susceptible to moisture and have less solubility than conventional glass-ionomers. They have less film thickness, preferable esthetic properties, and are easy to apply. These materials provide proper adhesion and have minor microfluidity (better resistance to marginal permeability). However, RMGICs are contraindicated for the fixation of more fragile all-ceramic constructions, as they expand due to water absorption, which can lead to the fracture of the restoration [57,58,59,60]. Excess can be removed from the marginal region of the restoration while in a gel state or after curing, whereas conventional GIC excess is recommended to be removed only after curing. These materials are available in the form of powder–liquid, paste–paste, and in the form of capsules [60,61].

### 3.5. Resin Cements

Resin cements are the most recent luting material developed for dental applications. During the early stages, resin cements failed due to high polymerization shrinkage and insufficient biocompatibility. Currently, resin cements have the ability to form a chemical bond with dentin and enamel and have higher bond strength and more predictability [62,63,64]. The bonding is usually achieved with organophosphonates, hydroxyethyl methacrylate (HEMA), or 4-methacryloxyethyl trimellitate anhydride (4-META) [62,65,66]. During polymerization, adhesive monomer-impregnated exposed collagen fibrils become entangled with them to create the hybrid layer and achieve high tensile bonding strengths [67]. HEMA enhances the penetration capability of dentinal substrates, and it has been shown that strong bond strength is achieved when the dentin is treated with HEMA, and the improvement of bond strengths is dependent upon the time period of HEMA application [68]. It shows the formation of a transitional zone of resin-reinforced dentin (hybrid layer) following pre-treatment with a 10-3 solution (10% citric acid/3% ferric chloride). The adhesive resin impregnates the exposed collagen bundles with which it entangles to create the hybrid layer, which is essential in the attainment of high tensile bond strengths. Hence, HEMA application to dentinal substrates enhances monomer diffusion and entanglement with dentinal components and facilitates the formation of hybrid layers.

Resin cements are composite materials with different chemical compositions. They consist of a resin matrix (e.g., Bis-GMA or urethane dimethacrylate) and fine particles of inorganic fillers. First of all, they differ from restorative composites by their low filler content (50–70% glass or silicon dioxide) and viscosity. In addition, there is a correlation between the amount of filler and the mechanical properties: the lower the number of fillers, the lower the mechanical strength [69,70,71].

Resin cements are insoluble and have superior mechanical and physical properties, as compared with other previous luting materials [62,65,66]. The clinical advantages of resin cements include high resistance to compression forces, low thermal expansion coefficients, high flexural strengths, and superior hardness when compared with other luting materials. In addition, resin cements are characterized by high fatigue strength, adhesion to many materials, the ability to modify shade and color, high retention, resistance to wear at the margin of the restoration, and low marginal permeability [72,73,74]. Resin cements provide an optimal bond with all-ceramic restorations and evenly distribute the compression force along all contact surfaces [69,72,73]. Resin cements are mostly used for the cementation of full-cast metal crowns, ceramic crowns, zirconia constructions, indirect composite restorations, traditional metal–ceramic constructions, metal and glass fiber post, implant-supported crowns and bridges, and ceramic veneers [16].

This material can be divided into adhesive or self-adhesive cements (Figure 1). When applying adhesive cements, the tooth should be previously acid-etched with phosphoric acid, followed by the adhesive system application. With total acid etching, the smear layer is eliminated and demineralization of the dentin occurs to a depth of 3–5 µm, exposing the collagen fibers [74,75]. In vital teeth, the adhesive is reported to penetrate about 10 µm into the dentin, forming a hybrid layer [76,77]. Dentin adhesives are thought to reduce the pulp reaction and reduce marginal micropermeability. Adhesive resin cements provide increased marginal sealing than zinc phosphate cements. However, the problem of the complete removal of excess cement from the hard-to-reach margins can hinder the application of resin cements to bond restorations with subgingival margins [76,78]. While using self-adhesive cements, acid treatment and the application of adhesives are not required, except for preparation in enamel, in which acid etching is still beneficial for increased bond strength values. Self-adhesive or self-etching resin cements present in their composition components that are able to promote, at the same time, bond strength to the substrate and restoration, for example, the presence of the MDP (10-Methacryloyloxydecyl dihydrogen phosphate) molecule. However, for glass ceramics, for example, the etching and silane layer is still necessary. Therefore, the instructions for use must be carefully checked before use. In addition, such cements have excellent mechanical and optical properties and provide strong adhesion to the tooth surface and other materials [79,80]. The term self-etching is also applied to specific adhesive systems, which eliminates the phosphoric acid etching step because an acidic component in the adhesive is responsible for this procedure. Most of the time, these adhesives are developed to be used only in association with the resin cements from the same manufacturer system. Therefore, this association promotes some benefits, such as faster polymerization and standardized procedures.

Resin cements are also classified according to the polymerization process: chemical-cure, light-cure, or dual-cure (Figure 1). Chemical or self-curing cements are polymerized due to a chemical reaction with peroxide as the initiator. Due to chemical components, self-curing resin cements have lower color stability; thus, they are not indicated to bond with translucent or thin ceramic restorations. For this, light-cure resin cements are used. Chemical polymerization materials harden slowly and gradually, causing less shrinkage stress. Light-cure cements are cured due to the activation of photoinitiators. Their main disadvantage is the controlled polymerization time when compared with self-curing materials [81,82], while dual-cure cements contain amine initiators (chemical) and photoinitiators (light) that allow the start of the polymerization process with the aid of a light source. Then, this light-curing reaction activates the chemical reaction that will happen in a long process. The catalyst in dual-cure cements promotes the final hardening of the cements in areas inaccessible to light after initial rapid light polymerization [83,84]. Dual cements present the advantage to be indicated for several clinical situations when the light intensity is compromised due to the restoration thickness or translucency. Thus, the final polymerization will be achieved due to the chemical reaction. Light-curing cements are indicated to cement ceramic or indirect composite restorations that are less than 1.5 mm thick and provide sufficient light penetration. Light curing may not result in adequate resin polymerization under thick zirconia structures. LED light sources should be preferred over QTH for curing dual-cure resin cements, especially for those under thicker zirconia restorations [85]. Dual-curing cements are recommended for ceramic and composite restorations with a thickness of 1.5–2.5 mm. The chemical cure of dual resin cements is sufficient to allow their use under zirconia and thick ceramic restorations [86]. Self-curing cements are used to cement the restorations that block light, such as zirconium oxide all-ceramic crowns and bridges, ceramic and composite inlays and onlays (>2.5 mm), adhesive fixed partial dentures, and metal constructions [87,88]. Some resin cement systems can activate the chemical polymerization of the adhesive system to avoid the need to cure it before cementation [89]. The use of dual adhesives is another important protocol during the cementation of prostheses [90]. Coelho Santos et al. [77] evaluated the influence of a dentin adhesive application technique (pre-curing vs non-pre-curing) on microtensile bond strength to dentin and adhesive layer thickness in indirect resin restorations. They found that when pre-curing the adhesive system before applying the cement, an adhesive layer was formed, while no adhesive layer was seen for the adhesives used without the pre-curing step. 

The disadvantage of resin cements is that they cannot prevent secondary caries compared to RMGIC, as the resin cements have fewer caries-inhibitory effects compared to RMGIC [91,92]. Some products have short working times, lack anti-corrosion activity, and generate hypersensitivity during polymerization shrinkage. In general, resin cements are less biocompatible than GICs. HEMA is known to be released from RMGICs and can have damaging biological properties, ranging from pulpal inflammation to allergic contact dermatitis and other immunological responses [93,94,95,96]. Another study found that the toxic effects are material-dependent; the different protocols for the application of these dental materials to dentin may interfere with their cytotoxicity [94]. Hence, care needs to be taken and dental personnel are at risk of adverse effects.

Moreover, for the successful clinical application of cements, proper handling is important. In addition, the success of resin cements is highly dependent on humidity control, as the film thickness in resin cements is more than that of other cement types. Therefore, during the luting procedure, studies have shown that applying pressure to restoration for up to 3 min improves marginal contact and bond strength [69,97,98,99]. Another issue is that, in case of necessity, the restoration is difficult to remove [69,99]. 

## 4. Provisional Luting Cements

Provisional cements protect the dental pulp while fixing provisional restorations. In some cases, even the final restoration can be temporarily cemented with a provisional luting agent in order to ensure pulp and periodontium health, giving to the patient the opportunity to evaluate the appearance and function of restoration [22,27,100,101].

The provisional cements should have the following qualities [16,30]:

- Easy to handle and spread;
- Sufficient strength to retain indirect restorations for a short-term period;
- Easy to remove and clean from the substrate and restoration;
- Optimal working and curing times;
- Sufficiently viscous and easy to apply;
- Biocompatible without damaging the soft tissues and the prepared tooth;
- Removed without releasing subproducts;
- Inert to the adhesive properties of the long-term cement.

Regardless of the aim, provisional cementation requires thorough monitoring. The removal of the restoration may cause some difficulties for long-term cementation—issues that can be prevented by using a small amount of Vaseline in the crown’s intaglio surface. Additionally, the luting material should be applied only to the restoration margins, allowing sealing and, at the same time, ensuring easy removal in the future [17,22,30,100,101,102,103,104,105,106]. On the other hand, adhesive and cohesive failures of the provisional restoration are always common complications. When the restoration is displaced, the patient experiences some discomfort, and caries lesions can develop in the unprotected dental tissue [22,102]. Provisional cementation should be performed only when the patient has been given clear explanations about the purpose and duration of it, as well as the importance of promptly communicating with the dentist in case of detachment [27,100,103]. Some cements contain fluoride to hinder hypersensitivity and chlorhexidine to protect the adhesive interface [30,104,105]. Some examples of temporary cements that can release fluoride are zinc-oxide-based temporary cements (Tempbond™ NE, TBNE, Kerr, Italy) and glass-ionomer cements (Ketac™ Cem, 3M ESPE, Seefeld, Germany) [107,108]. The addition of fluorides slightly increases the solubility of the cements.

In addition to the ideal characteristics, provisional cements should not inhibit the polymerization of the impression material, be radiopaque, or have low solubility, and they should look natural and have no taste or smell.

## 5. Substrate and Restoration Surface Treatments before Cementation

Regardless of the selected luting agent, the effectiveness of cementation procedures reduces if the material comes into contact with moisture, blood, or saliva. Therefore, thorough cleaning and drying of the substrate and restoration are mandatory; meanwhile, overlying the tooth should be avoided. In case non-adhesive cements are used, the tooth should be cleaned (it is recommended to use a pumice stone and/or chlorhexidine), thoroughly dried, and coated with a special cavity varnish or dentin bonding resin [109]. In the case of adhesive cements, the substrate surface should also be cleaned and treated according to the cement system protocol.

Surface treatments of the restoration increase the microtensile bond strength, considering the restoration surface treatment will vary according to the restorative material composition [110,111]. At present, various surface treatments for ceramics are available for better bonding to tooth structure [112,113,114]. Ceramic restorations also require a sandblasting procedure followed by ceramic primer application. Hydrofluoric acid (HF, 10%) etching should be used for the surface conditioning of the studied hybrid ceramic to have increased microtensile bond strength between resin cement and a hybrid ceramic [112,114]. It was found that air abrasion followed by universal adhesive and HF acid followed by silane application appears to be the best strategies for glass ceramics to optimize the bond strength [115].

Indirect composite restorations need sandblasting; however, this treatment should be followed by an adhesive layer. Metallic restorations require a sandblasting procedure followed by a metal primer application. The surface treatment of gold restorations can be performed by heat treatment, alumina blasting, and tin plating [116]. Heat-treated gold alloys show more resistance to bond failure than alumina blasting or tin plating.

## 6. Optical Properties of Luting Cements

Opaque posts may affect the esthetics of all-ceramic single-unit crowns, as the thin layers of luting cement may not be sufficiently opaque. Vichi et al. [117] studied the influence of opaque posts (carbon fiber and zirconia) and esthetic posts and the shade and thickness of luting cements on the esthetics of all-ceramic restorations. The final esthetic result of the all-ceramic glass–ceramic restoration was not affected by the presence of different substrates with different colors for >2.0 mm crown thickness. For a ceramic thickness of 1.5 mm, color differences decreased, and most differences were appreciable only with laboratory instruments. When ceramic thickness was 1 mm, all other variables were visually appreciable, and the use of a full ceramic crown is contraindicated because color matching of the abutment is required to ensure an acceptable esthetic result. Hence, the differences in cement thickness (0.1 or 0.2 mm) may slightly affect the final result. The cement shades allow only minor esthetic corrections, which might be instrumentally detectable but are clinically not relevant.

The resin cements still have a higher contrast ratio and lower translucency [118]. Chang et al. [119] studied the optical properties of resin-based composite cements and assessed their effects on the color of ceramic crowns (Empress and Kantana). They found that the cements created perceptible color differences with particular combinations of die material and cement and ceramic crowns. In the cervical region, the color changes were affected by the dark brown abutment but could be reversed with bleach luting cement; in the body region, this was true only for the Empress crown. Neither different abutment colors nor different luting cement shades resulted in perceptible color changes in the incisal regions. Furthermore, various shades of resin cements may adversely affect the final color of translucent restorations, especially veneers [120,121]. The type, shade, and thickness of resin cement and the shade of the ceramic all influenced the resulting optical color of veneer restorations.

Like resin cements, GICs are also available in various shades. Generally, GICs are less opaque compared to zinc phosphate and zinc polycarboxylate cements; they still have a higher contrast ratio and lower translucency [122]. Zinc phosphate cements and zinc polycarboxylate cements are also opaque as they have a higher contrast ratio and lower translucency.

## 7. Conclusions

The long-term outcome of prosthetic treatments depends on the condition of periodontal tissues, the choice of prosthetic constructions, and the fabrication technology. The selection of the luting agent should be guided depending on the case, considering the duration of the bonding procedure, substrate, and restoration type and material. The manufacturer’s instructions for the thin cement-layer thickness are important for the long-term success of the restoration. Currently, all luting cements still have a higher contrast ratio and lower translucency. It is important to distinguish the mechanical properties and general characteristics of luting materials in order to identify the best options for each case. At present, the most widely used cements are glass-ionomer and resinous luting agents.

Proper surface treatment protocol for both the substrate and restoration surfaces is needed before cementation. Surface treatments of the restoration increase the microtensile bond strength. For glass ceramics, air abrasion followed by universal adhesive and HF acid followed by silane application appear to be the best strategies. The surface treatment of indirect composite restorations can be performed with sandblasting. Metallic restorations require a sandblasting procedure followed by a metal primer application. Gold restorations can be performed by heat treatment, alumina blasting, or tin plating.

## Figures and Tables

**Table 1:** General characteristics of non-adhesive and adhesive cements.

Non-Adhesive Cements	Adhesive Cements
➢ Retention is mechanical. ➢ Need long axial walls of the preparation with slight taper (approx. 6°). ➢ Precise fit of the restoration (approx. 30–100 μm). ➢ Bond strength should be higher than the stress during chewing. ➢ The tooth is cleaned (recommended to use a pumice stone and/or chlorhexidine), thoroughly dried, and coated with a cavity varnish or dentin bonding resin.	➢ Retention is micromechanical and chemical; adhesive bonding between restoration and tooth. ➢ Bond strength is dependent on the stable adhesive interface and bondable materials (tooth surface, restoration, and cement). ➢ Adhesive fixation is also conditioned by environmental control (the absence of moisture). ➢ Bond strength should be higher than the stress during chewing. ➢ The substrate surface is cleaned and treated according to the cement system protocol.

**Table 3:** Indications, retention, advantages, and precautions of dental luting agents [16,22,27].

Luting Agents	Indications	Adhesion	Excess Removal (I *: Easy)	Retention (I *: High)	Advantages	Disadvantages	Precautions
Zinc phosphate	1,3,6,9,10	Chemical	Easy	Moderate	History of use	Solubility, leakage	Use for “traditional” cast restorations
Zinc polycarboxylate	1,3,4,6	Chemical	Medium	Low/moderate	Biocompatibility	Low strength, solubility	Do not reduce powder/liquid ratio
Glass-ionomer ^F^	1,3,6,9,10,12	Chemical	Medium	Moderate to high	Translucency	Solubility, leakage	Avoid early moisture exposure
RMGIC ^F^	1,3,9,10	Micro-mechanical	Medium	High	Low solubility, low microleakage	Water sorption, history of use	Avoid with ceramic restorations
Resin cement	1–3, 5–12	Mechanical	Medium to difficult	High	Adhesive, low solubility	Film thickness, history of use	Moisture control

1—full metallic and metal–ceramic crown and partial FDP, 2—crown or partial FDP with poor retention, 3—MCC with porcelain margin, 4—casting on the patient with a history of post-treatment sensitivity, 5—pressed, high-leucite, ceramic crown, 6—slip-cast alumina crown, 7—ceramic inlay, onlay, and veneer, 8—resin-retained partial FDP, 9—cast post and core (metal), 10—aesthetic postcore and core (fiber and ceramic), 11—ceramic veneer, and 12—full zirconia and zirconia-based ceramic restorations. I *—ideal, FDP—fixed dental prosthesis, MCC—metal–ceramic crown, ^F^—fluoride release, and RMGIC—resin-modified glass-ionomer cement.

## Data Availability

Not applicable.
